# Tinea Blepharociliaris: A Case Report and Literature Review

**DOI:** 10.7759/cureus.76308

**Published:** 2024-12-24

**Authors:** Jesús Iván Martínez-Ortega, Jacqueline E Mut Quej, Tiffany Karoly Medina Angulo

**Affiliations:** 1 Dermatology, Dermatology Institute of Jalisco, Zapopan, MEX; 2 Histology, Autonomous University of Nuevo Leon, Monterrey, MEX; 3 Internal Medicine, Regional General Hospital No. 12 Lic. Benito Juárez, Yucatán, MEX; 4 General Practice, Autonomous University of Campeche, Campeche, MEX

**Keywords:** antifungal treatment for eyelid infections, atypical tinea infections, dermatophyte eyelid infection, madarosis, ocular dermatophyte infection, periocular fungal infection, tinea blepharociliaris, tinea faciei, tinea periocular, trichophyton rubrum

## Abstract

Tinea blepharociliaris is a rare dermatophyte infection affecting the eyelashes and eyelids, often misdiagnosed as blepharitis, eczema, or bacterial infection, leading to ineffective treatments and recurrent symptoms. We report a case of a 10-year-old girl with erythematous plaques and fine scaling on the eyelids and eyelashes, initially suspected to have facial tinea or contact dermatitis. Direct mycological examination confirmed the presence of fungal filaments and spores, with culture identifying *Trichophyton rubrum* as the causative organism. Systemic and topical antifungal therapy resulted in complete resolution. Tinea blepharociliaris, though uncommon, mimics other periocular conditions, especially when modified by prior corticosteroid use. Differential diagnoses include primary and secondary causes of eyelash loss, such as chronic blepharitis and preseptal cellulitis. Clinicians should consider systemic antifungals for periocular dermatophyte infections involving hair-bearing areas, similar to the approach for tinea barbae and tinea capitis. Proper categorization, diagnosis, and treatment are essential to prevent misdiagnosis and ensure successful outcomes.

## Introduction

When dermatophyte infections affect the face, they are referred to as tinea faciei (facial ringworm, also known as tiña facial in Spanish) [1]. Dermatophytes are fungi with a strong affinity for keratin, the main protein found in skin, hair, and nails [2]. These fungi are the primary cause of tinea infections, and they have recently been reclassified into nine genera, with *Trichophyton, Epidermophyton, Nannizzia, *and *Microsporum* being the most prevalent [3]. Compared to tinea corporis or other body locations, tinea faciei often presents atypically, particularly when steroid use has altered the appearance of the infection [1,4].

When dermatophyte infections involve the eyelids, the condition is called tinea periocular, and when the eyelashes are affected as well, it is referred to as tinea blepharociliaris [5]. This condition is characterized by eyelash loss (madarosis), erythema, and scaling [5]. Madarosis specifically refers to the loss of eyelashes or eyebrows, whereas alopecia is a broader term used to describe hair loss from any hair-bearing skin, which can result from various infectious, inflammatory, or systemic conditions. Despite the functional and psychological significance of these features, this condition is often underrecognized, understudied, and commonly misdiagnosed [6].

Due to its atypical presentation, tinea blepharociliaris is frequently mistaken for conditions such as eczema or bacterial infections, resulting in improper treatment and recurrent exacerbations [6].

This report highlights a case of tinea blepharociliaris, emphasizing the importance of considering this condition in the differential diagnosis of periocular involvement. It also serves to underline the need for accurate diagnosis to ensure effective management and prevent recurrence.

## Case presentation

**Figure 1:**
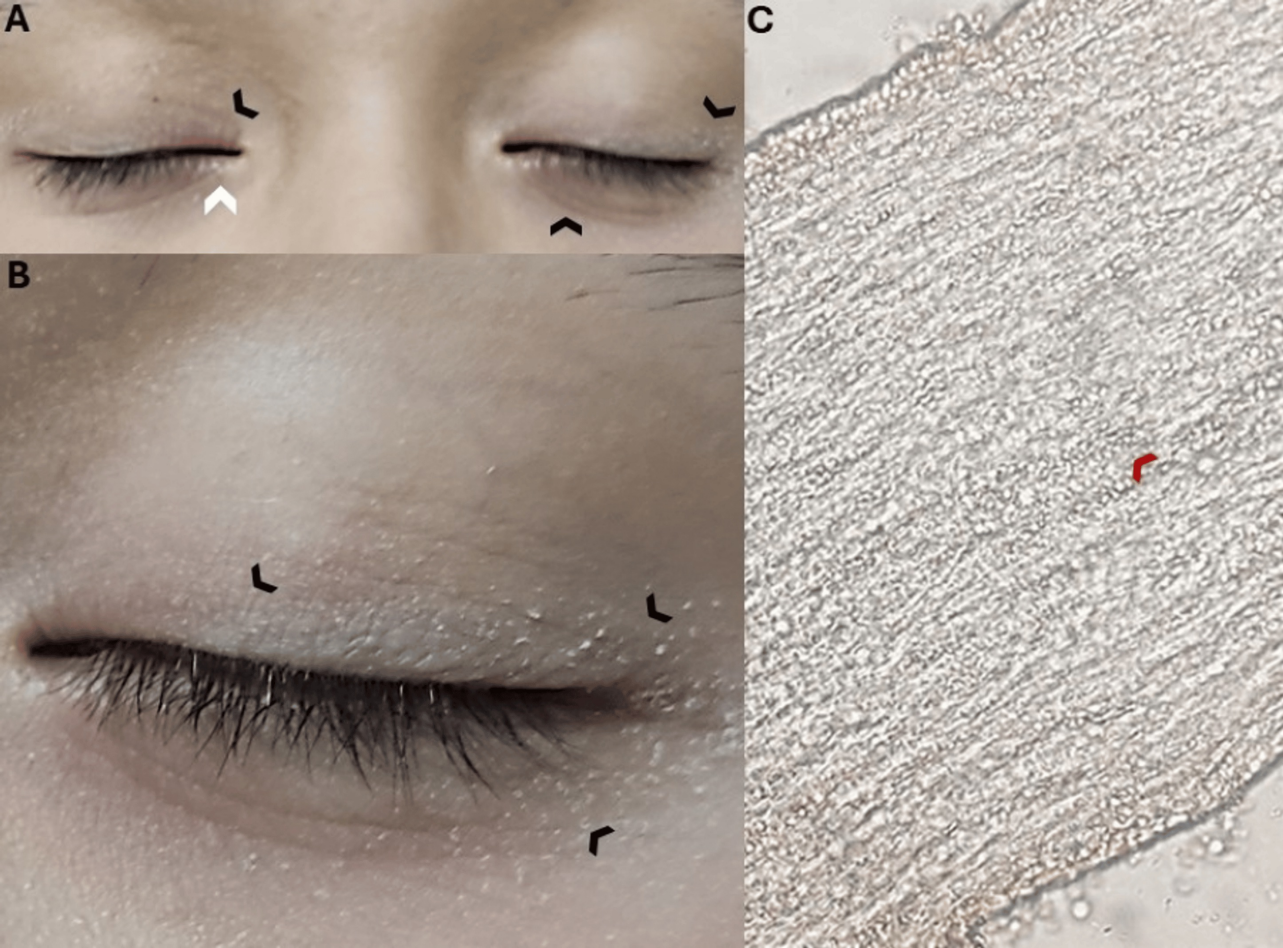
Clinical and Direct exam findings in a 10-year-old female with tinea blepharociliaris. (A) Frontal view of both eyelids showing mild erythematous plaques with fine scaling along the eyelid margins (black arrows), predominantly affecting the upper eyelids. Slight madarosis is noted on the right lower eyelid (white arrow). (B) Close-up of the left eye demonstrating scaling along the eyelid margin (black arrows), with associated erythema and slight edema, particularly around the base of the lashes. (C) Potassium hydroxide (KOH) 20% preparation showing the presence of fungal hyphae (filaments) and arthroconidia (spores) (red arrow), consistent with a diagnosis of dermatophyte infection, observed at 200x magnification. The test was performed on skin scrapings containing hairs and scales from the affected eyelid, with no need for hair plucking.

A 10-year-old female patient with no significant medical history presented with a one-month history of lesions on her eyelashes and eyelids. She reported no use of cosmetics, creams, or chemical substances, and no contact with pets. Physical examination revealed mildly erythematous plaques with fine scaling along the anatomical contours of both eyelids. Differential diagnoses included dermatophyte infection, contact dermatitis (despite the absence of any clear exposure history), and blepharitis. A direct mycological examination with potassium hydroxide (KOH) (Figure 1) and fungal culture were performed.

**Figure 2:**
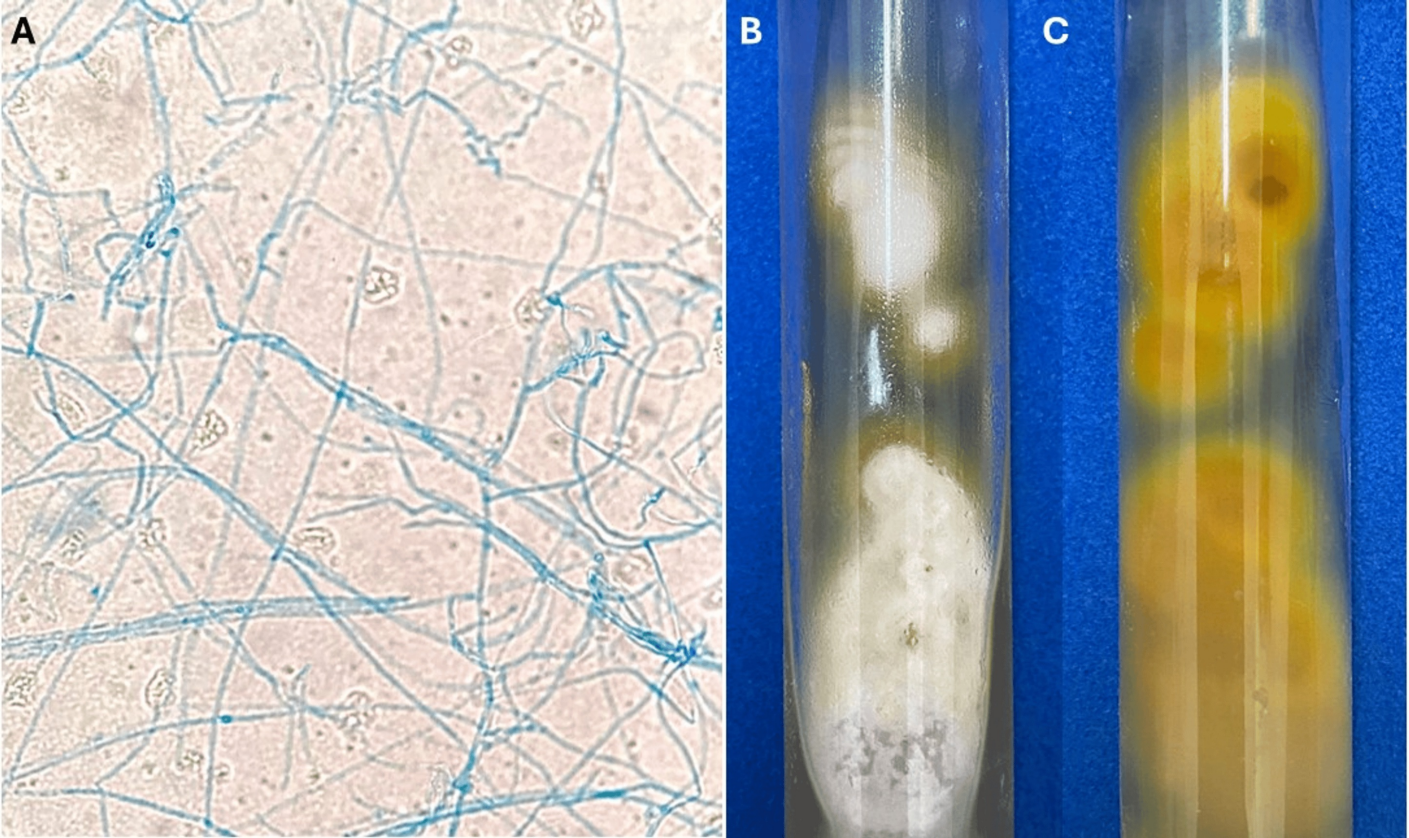
Results of Mycological Examination Laboratory findings in a 10-year-old female with tinea blepharociliaris. (A) Microscopic view of a lactophenol cotton blue stain, showing septate hyphae and arthroconidia, characteristic of dermatophyte infection, observed at 200x magnification. (B) Sabouraud dextrose agar culture with white, cottony colonies displaying a powdery texture, typical of *Trichophyton rubrum*. (C) The reverse of the culture plate shows yellowish-brown pigmentation, confirming *Trichophyton rubrum* as the causative organism.

The KOH examination revealed fungal hyphae (filaments) and arthroconidia (spores), leading to the initiation of systemic and topical antifungal therapy. The patient was prescribed oral terbinafine 125 mg daily for one month, along with 1% ketoconazole cream applied twice daily for one month, followed by twice-weekly applications for an additional two months. Fungal culture confirmed *Trichophyton rubrum* as the causative agent of facial tinea (Figure 2). At the one-month follow-up, the patient reported complete resolution of her lesions.

## Discussion

Tinea faciei, an uncommon superficial dermatophyte infection of the glabrous skin on the face, accounts for roughly 2% of all tinea cases. In comparison, tinea corporis reaches 15% of cases in the same study [1]. The causative agents of tinea faciei vary by geographic region and environmental factors, with common pathogens being *Microsporum*, *Trichophyton*, and *Epidermophyton *species [1,5].

A study analyzing atypical presentations of tinea found that 31.2% (48 out of 154) of cases were tinea faciei, indicating a higher prevalence of atypical presentations on the face compared to other body regions [7]. In another study focused specifically on tinea faciei, 57.1% of patients (61 out of 107) presented with typical forms, while 42.9% (46 patients) displayed atypical forms [8]. It is estimated that up to 70% of patients with tinea faciei are initially misdiagnosed with other dermatoses [9,10]. For periocular tinea, a study involving 10 cases reported a misdiagnosis rate of 100% [11].​​​​​​** **However, this finding should be interpreted with caution due to the extremely limited sample size, which precludes generalization to larger populations. In this study, misdiagnosis or delayed recognition of periocular tinea resulted in significant diagnostic delays, lasting a median of 6 weeks, with some cases extending up to 24 weeks, thereby prolonging patient discomfort and complicating treatment [6,11].

Tinea blepharociliaris, a fungal infection of the eyelashes and eyelids, is an underreported condition with limited epidemiological data [6]. To address this gap, we conducted a comprehensive literature search in MEDLINE and Google Scholar, using terms such as 'tinea blepharo-ciliaris,' 'tinea ciliaris,' and 'tiña blefarociliar' (in Spanish). This search aimed to capture the clinicoepidemiological data associated with these terminologies and provide a thorough review of reported cases, as summarized in Table 1 [6,10,12-18]. To ensure the accuracy and relevance of the data, we excluded three cases that lacked clear documentation of hair involvement [19-21]. 

**Table 1:** Summary of reported cases of tinea blepharociliaris in the literature Overview of published cases of tinea blepharociliaris, including demographic details, clinical presentation, identified pathogens, treatment, and outcomes. This table was prepared by Jacqueline E. Mut Quej and is a modification of the data presented by Wang et al. [16], with additional cases included. Cases in the table are derived from the references [6,10,12–18]. Abbreviations: KOH, potassium hydroxide

Author	Year of Publication	Country	Age	Gender	Exposure and Risk Factors	Symptom History	Eyelid and Eyelash Involvement	KOH Test Results	Identified Pathogen	Treatment	Outcome
Creach et al. [12]	1995	France	48	Male	Cat contact	Unilateral blepharitis, resistant to topical antibiotics and steroids	Broken eyelashes and scaly annular lesions of unilateral eyelid	Spores	Microsporum canis	Griseofulvin (1 g daily) for 6 weeks	Complete remission
Machado et al. [13]	2004	Brazil	22	Female	Not mentioned.	Scaly ring form lesion on the left eyelid for 2 months, resistant to steroids and calcipotriol.	A scaly ring shape lesion on the right eyelid.	Septated hyaline hyphae	Microsporum gypseum	Itraconazole (200 mg daily) for 2 weeks.	Complete remission
Sahin et al. [14]	2014	Turkey	69	Male	Not mentioned	Itching, stinging, eyelash loss, conjunctivitis for 10 years, resistant to antibiotics and steroids	Eyelash loss, scales on margins of both upper and lower eyelids	Ectothrix	Microsporum audouinii	Itraconazole (100 mg daily) for 3 months	Complete remission
	2014	Turkey	40	Female	Not mentioned	Eyelids itching, stinging, foreign body sensation for 4 years, resistant to steroids	Scales on the eyelid margin	Ectothrix	Trichophyton verrucosum	Itraconazole (100 mg daily) for 3 months	Complete remission
Buruianca et al. [15]	2015	Romania	24	Male	Shepherd living in a rural environment	Right eyelid swelling for 2 weeks, refractory to systemic antibiotics	Right upper and lower eyelid edema with purulent secretions and massive hair loss	Ectothrix	Trichophyton interdigitale, zoophilic strain	Itraconazole (100 mg twice daily) and fluconazole eye drops 0.3% for 4 weeks	Complete remission
Wang et al. [16]	2016	Taiwan	13	Female	Kept two rabbits, diabetes mellitus	Right eyelid swelling, itching for 2 weeks	Right upper and lower eyelid edema with pustules, broken eyelashes, black dots	Endothrix	Trichophyton benhamiae	Griseofulvin (500 mg daily) and clotrimazole cream for 4 weeks	Complete remission
Verzı et al. [17]	2020	Italy	6	Male	Not mentioned	8-week history of an itchy rash of the left eye that was treated with topical antibiotics and corticosteroids without improvement.	Erythematous and desquamative patch involving the eyelid and the periocular area. The upper eyelashes appeared broken at different levels. Trichoscopy examination of the eyelashes: diffuse scaling, broken hairs, bent hairs, and Morse code hairs.	Ectothrix	Microsporum canis	Griseofulvin (150 mg daily) for 4 weeks.	Complete remission
Zhang et al. [18]	2020	Brazil	10	Male	Dog contact	2-week itchy erythema on periocular skin and multiple papulopustular on the left upper palpebra	Erythema with mild scales on the periocular skin and several sesame-sized milky papulopustules on his left upper palpebra.	Septated hyphae.	Trichophyton rubrum	Itraconazole (100 mg daily) for two weeks and topical bifonazole cream once every night for four weeks.	Complete remission
Shimoyama et al. [10]	2022	Japan	38	Female	Not mentioned.	A 3-week history of an itchy, solitary erythematous lesion on the left medial angle of the eyelid resistant to topical corticosteroid.	Pruritic, annular, scaly erythematous patch approximately 2.5 cm in diameter, growing centrifugally on the medial angle of the left eyelid.	Ectothrix	Nannizzia gypsea	Itraconazole (100 mg/day) for 8 weeks.	Complete remission
González-Escalante et al. [6]	2023	México	7	Female	Not mentioned	The pruritic and alopecic area on the upper eyelid of the left eye is resistant to antibiotics and steroids for 15 days.	Alopecia on the upper eyelid of the left eye	Ectothrix	Trichophyton mentagrophyte/T. interdigitale complex	Ketoconazole shampoo 2% and itraconazole (3 mg/kg/day) for 3 weeks	Complete remission

Notably, many cases of tinea blepharociliaris may be underdiagnosed due to insufficient examination of the eyelashes and eyelids. As suggested by other authors, the scarcity of reported cases is more likely attributable to underdiagnosis rather than neglect. This oversight has significant implications, as the diagnosis, treatment, and prognosis differ from other facial infections [6]. For example, cases of tinea faciei affecting the eyelids might involve hair parasitization if thoroughly evaluated [10]. Similar conditions may appear under alternative terms like 'tinea orbitalis', 'eyelid tinea with blepharitis', 'tinea blepharitis,' 'tinea orbitalis' and 'dermatophytic blepharitis' though without hair assessment [11,19-21]. Some authors suggest reserving 'tinea periocular' for cases where only eyelashes, not eyelids, are affected [5]. Accurate classification and intentional assessment for hair parasitization in tinea periocular and tinea blepharociliaris are essential, similar to practices for tinea barbae or tinea capitis, which also involve hair-bearing areas [1,4,6].

**Figure 3:**
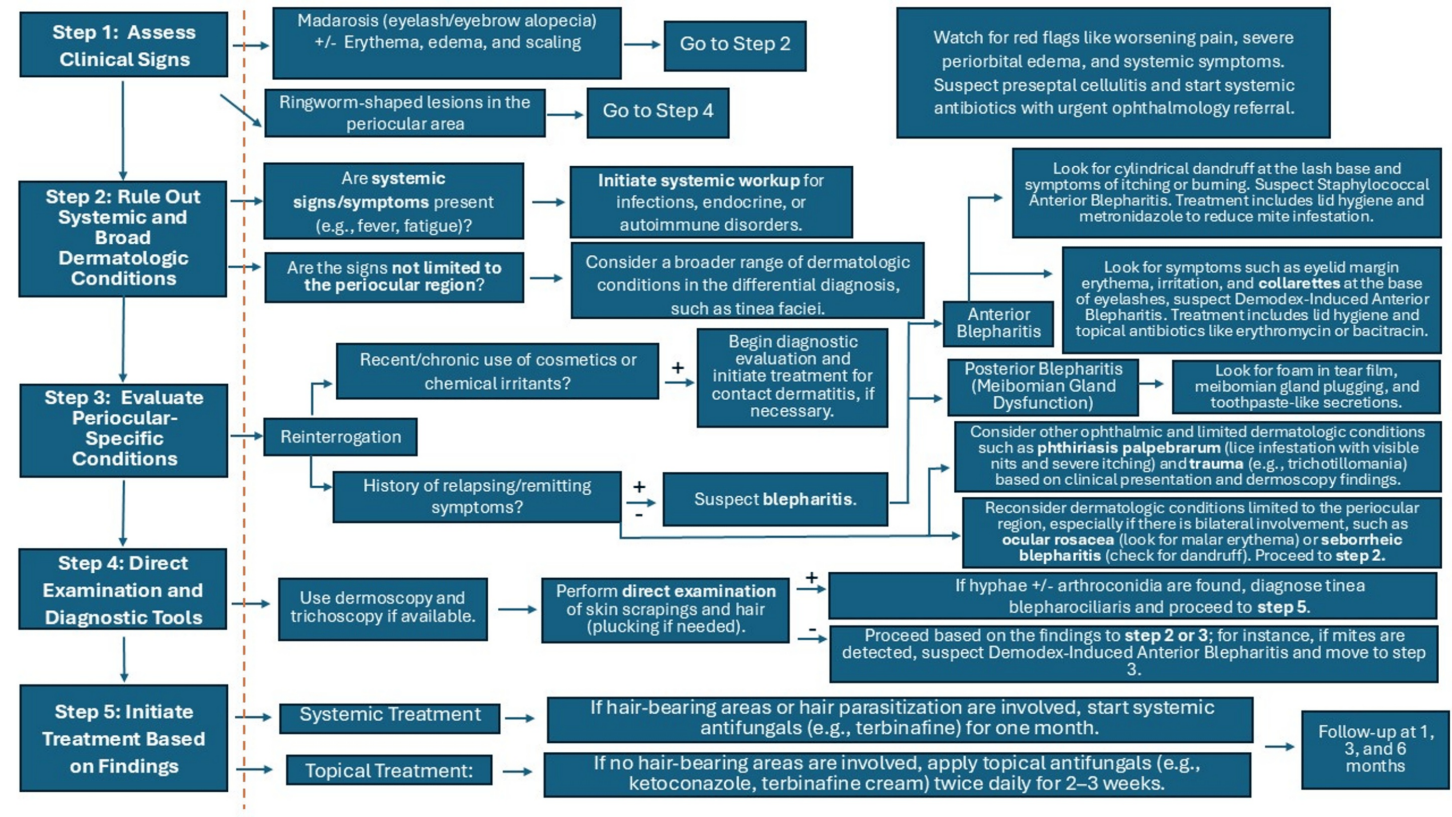
Diagnostic algorithm and management considerations for tinea blepharociliaris, including differential diagnoses and follow-up recommendations Flowchart illustrating the diagnostic algorithm for tinea blepharociliaris and its differential diagnosis. The algorithm begins with a clinical assessment of periocular symptoms, such as pruritus, erythema, scaling, and madarosis (loss of eyelashes). Key diagnostic steps are positioned to the left of the punctuated yellow line, including direct and dermoscopic examination. While culture is not required to initiate treatment, it is recommended to document and morphologically identify the species. To the right of the yellow line, differential diagnoses, including seborrheic dermatitis, Demodex infestation (blepharitis), bacterial blepharitis, phthiriasis palpebrarum, and allergic contact dermatitis, are outlined with distinguishing clinical features and recommended diagnostic approaches. The flowchart highlights the importance of systemic antifungal therapy for hair-bearing areas due to the risk of deeper fungal invasion and the need for the treatment to penetrate hair shafts and follicles effectively. For non-hair-bearing regions, topical antifungal treatment may be appropriate. Additionally, follow-up at 1, 3, and 6 months is emphasized to monitor treatment response, identify potential relapses, and ensure complete resolution of the infection. Figure credit: Tiffany Karoly Medina Angulo and Jesús Iván Martínez-Ortega created this flowchart.

Clinically, in addition to typical signs such as inflammation, erythema, and scaling, alopecia of the eyelashes and/or eyebrows (madarosis) should be considered, even in cases with minimal signs and symptoms. The differential diagnosis for this condition is broad, encompassing both primary and secondary causes of madarosis. Primary dermatoses include atopic dermatitis, seborrheic dermatitis, seborrheic pemphigus, and psoriasis, while secondary causes may involve infections (e.g., syphilis, leprosy, herpes zoster), nutritional deficiencies, endocrine disorders, autoimmune diseases, and genetic conditions. Notably, conditions such as atopic dermatitis, eczema herpeticum, seborrheic dermatitis, psoriasis, and seborrheic pemphigus may also exhibit erythema and scaling. However, they are generally distinguishable by the extent of the affected area [5,6,22,23]. Given its high frequency and typically localized nature, blepharitis (either anterior or posterior) and contact dermatitis should be the primary differential diagnoses. In contrast, diagnosing preseptal cellulitis can sometimes result in unnecessary overtreatment [11,24]. Other less frequent but noteworthy secondary causes include trichotillomania, demodicosis, and phthiriasis palpebrarum (Figure 3) [5,24,25].

Trichoscopy has been proposed as a valuable diagnostic tool for tinea blepharociliaris, especially when evaluating hair-related signs [5]. Wood's lamp examination can also aid in identifying cases caused by *Microsporum canis*, though it is effective in only a minority of cases [26]. Direct examination of scales and hair for mycological testing should be conducted whenever available. In settings where mycological testing is not accessible, clinical evaluation is generally sufficient to start empirical treatment. In such cases, systemic empirical treatment should be based on the affected region, even if hair parasitization is not documented, as recommended by other authors [5].

While tinea faciei affects glabrous skin, tinea blepharociliaris and tinea periocular involve hair follicles and should be managed similarly to tinea barbae and tinea capitis. This distinction informs diagnostic and treatment approaches, including the use of trichoscopy and hair sampling. Systemic antifungals, such as griseofulvin (10-20 mg/kg/day for 8-12 weeks) or terbinafine (10 mg/kg/day for adults, or 3-6 mg/kg/day for children under 40 kg), are recommended due to hair follicle involvement [4-6,27,28].

Follow-up is crucial, as up to 30% of patients fail to achieve a complete cure, and relapse rates of up to 22% have been reported within three months for tinea infections. For these reasons, early follow-up at one month, along with intermediate follow-ups at two to three months and at six months, may be reasonable to ensure effective management and reduce the risk of recurrence [28].

## Conclusions

Eyelids and eyelashes, as hair-bearing areas, require specific attention in the diagnosis, categorization, and treatment of fungal infections like tinea blepharociliaris. This condition is often misdiagnosed as other periocular disorders, ranging from mild conditions like chronic blepharitis to more serious ones such as preseptal cellulitis. Such misdiagnoses can lead to delayed or overtreatment, recurrent symptoms, and prolonged patient discomfort. Accurate diagnosis is crucial and should include trichoscopy, mycological examination, or clinical suspicion and prompt empirical systemic treatment when diagnostic tools are unavailable.
